# Does the Implementation of Ride-Hailing Services Affect Urban Road Safety? The Experience of Madrid

**DOI:** 10.3390/ijerph19053078

**Published:** 2022-03-05

**Authors:** María Flor, Armando Ortuño, Begoña Guirao

**Affiliations:** 1 Department of Civil Engineering, University of Alicante, 03690 San Vicente del Raspeig, Spain; arorpa@ua.es; 2 University Institute of the Water and the Environmental Sciences, University of Alicante, 03690 San Vicente del Raspeig, Spain; 3 Department of Transport Engineering, Regional and Urban Planning, Universidad Politécnica de Madrid UPM, 28040 Madrid, Spain; begona.guirao@upm.es

**Keywords:** ride-hailing, Uber, road traffic injuries

## Abstract

In recent years, changes have occurred in consumption, ownership, and social relations, giving rise to new economic models in which technology enables new ways of connecting, creating, and sharing value. The nature of transport has transformed with the emergence of mobile applications, such as Uber and Cabify, which offer an alternative to the services traditionally provided by the taxi and chauffeur-driven hire vehicle (CDV) sectors. These services have developed within a context of market regulation of the taxi and CDV which are subject to considerable unjustified restrictions for entering and operating in the market, including the *numerus clausus* of licenses, the limited geographical scope of the license and, in the case of taxis, the regulation of prices as inflexible public rates. Bearing in mind the latest legislative changes affecting mostly the provision of the services of these platforms, this study analyzes whether the number of traffic accident victims has fallen since the introduction of these services in the city of Madrid using a Random Effects Negative Binominal model. The results show that the deployment of these platforms is associated with a reduction of 25% in the number of serious injuries and deaths.

## 1. Introduction

The advance of the sharing economy and collaborative consumption has had a strong impact on different sectors, particularly transport, with the emergence of companies such as Uber or Cabify which, using the p2p networks, have established a foothold in the complex sector of passenger transportation. This situation has generated an important debate and consequences for the classic passenger transport sectors which do not welcome the entrance of these companies into the market and particularly oppose the fact that they operate outside of any regulatory framework [1].

In Spain, Uber and Cabify operate through drivers with a CDV license (chauffeur-driven vehicle). These licenses are authorizations that permit the rental of chauffeur-driven vehicles, as their name indicates, and which have historically been used by chauffeurs, limousine drivers, or private transport services from/to the airport.

One of the most salient aspects of these authorizations is their limitation, as new licenses cannot be granted if there are more than one CDV for every 30 taxis [2]. Currently, this ratio of 1:30 is not respected, and in recent years, this has constituted the principal reason for the protests by the taxi drivers who demand that limits are set on the granting of CDV licenses. For this reason, in April 2018, the Royal Decree Law 3/2018 of 20 April was passed, regarding the leasing of chauffeur-driven vehicles with the principal novelty being the legal consolidation of a limit of one CDV license for every 30 taxi licenses. However, for the taxi drivers, these measures were “insufficient and poor” and the protests from this sector continued for months, leading to the collapse of the principal roads of Madrid and Barcelona for days and even violent incidences. Finally, the government agreed to negotiate a new legislative reform: the Royal Decree Law 13/2018 of 28 September. The existence of this regulation is justified, as it states that “*During the months after the entry into force of Royal Decree-Law 3/2018 of 20 April, it has become evident that the measures contemplated in said Royal Decree-Law were not sufficient to address the mobility, traffic congestion and environmental problems caused by the high increase in the supply of urban transport by way of passenger cars in the principal city centres of our country. Likewise, the rapid growth of this mode of transport could give rise to an imbalance between supply and demand for passenger vehicle transport, leading to an overall deterioration of the services, in detriment to the passengers*” [3].

However, before taking these decisions, the users were not consulted as to whether they felt that a greater supply, a better service, or lower prices negatively affected them. Moreover, the impacts of these services in terms of road safety, traffic congestion, and emissions have not been analyzed in the cities in which they operate. Actually, one of the current needs of smart cities is road safety. Cities want their citizens to be safe on foot, bicycle, or in their cars. Therefore, sustainable mobility plays a prominent role in this change. Smart mobility services, such as Uber or Cabify, are usually promoted by their suppliers as a fundamental component of a future sustainable transport. However, they are also associated with risks, such as an increase in congestion and inequality. Therefore, state intervention is essential to mitigate these risks and direct smart mobility in a way that contributes to achieving a sustainable transport [4], where the legislation and socio-technical context play a very important role for sustainable mobility [5].

In relation to the impacts of these platforms, there is evidence that the entrance of Uber and Lyft (its principal competitor in the United States) has had a positive environmental impact, thanks to the reduction in the ownership of vehicles and emissions [6,7,8]. On the contrary, other studies have suggested that the entrance of these new operators put new vehicles into circulation, giving rise to an increase in energy consumption [9], congestion [10,11,12], and the kilometers travelled by the vehicles [13]. However, George and Zafar [14] observed that these results depended on the context of the analysis. Meanwhile, the study focusing on urban cities of the United States by Li et al. [15] found that these services significantly increased traffic congestion in compact areas. Nevertheless, there is little evidence indicating that shared transport services reduce traffic congestion in expanding urban areas.

With respect to road safety, the majority of studies have focused on cities in the United States, except for the studies carried out in Chile, South Africa, and Great Britain [16,17,18]. The results obtained are contradictory, as some studies observe a decrease in driving under the influence of alcohol, while others find evidence that the entrance of these services has had no effect or has even increased fatal accidents [19,20,21,22,23,24,25,26]. However, the United States is an exceptional country with respect to traffic accident deaths. The Organization for Economic Co-operation and Development (OECD, Paris, France) data reveal that the traffic death rate per million inhabitants in the United States in 2018 (112.5) vastly exceeded that of the great majority of Western countries. For example, the rate of the United Kingdom was 27.6 per million inhabitants, that of Germany 36.6, and that of Spain 38.6 [27]. Therefore, although there was a reduction in traffic fatalities after the arrival of Uber to American cities, it is possible that a similar relationship does not exist in other countries which, in general, have a much lower risk of traffic fatalities. In fact, in those countries with much lower traffic accident rates than those of the United States, any added traffic congestion due to a higher number of car journeys could increase the risk of accidents instead of reducing it [18].

In short, and despite the huge expansion of Uber (with a presence in more than 60 countries throughout the world), there are very few studies on the implications of Uber on deaths caused by traffic accidents, and, particularly, in the case of victims with minor injuries, and the majority of these studies have focused on American cities. For this reason, this study, motivated mainly by the legislative changes that have affected these types of services in recent years in Spain (on which no previous analyses have been made), examines the implication of these services on the severity of traffic accidents in the city of Madrid. If the results are positive, and these types of services reduce deaths due to traffic accidents in Madrid, promoting their use could be advantageous for the city. However, on the contrary, if these services increase the risk of traffic accidents and deaths, it would be advisable to be cautious when granting new licenses and regulating these services, as is the case in Spain to date.

## 2. Materials and Methods

With a population of more than three million inhabitants, Madrid is characterized by the absence of territorial and social homogeneity. For many years, the urban sociology of the Spanish capital has been defined by two distinctive features of this lack of homogeneity: the differences between the north and south; and between the “center” and the “periphery”. Moneo [28] is one of the many studies carried out in the 1980s referring to the spatial segregation of the capital. The majority of these studies affirm that if you lived in a southern or south-eastern district outside of the “border” of the M-30 you were highly likely to live in a “segregated” neighborhood or district. In fact, if a line was drawn from north-east to south-west (to simplify, between the A-2 and A-5 roads), those districts and neighborhoods to the south of this line (districts in the south and south-east of the city, although with some exceptions, such as Tetuán and neighborhoods such as Embajadores in the Central district) were technically, defined as segregated districts and neighborhoods. The second characteristic related to the differences existing between the central core, the neighborhoods, and districts inside the M-30 and the “peripheral districts” [29].

**Figure 1:**
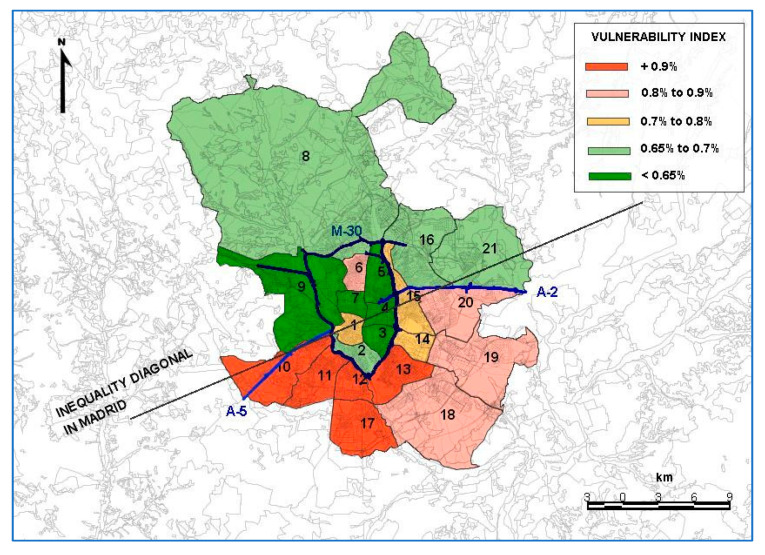
Geographical and social differences in Madrid. Source: Own elaboration, based on the document “Metodología para la elaboración del Índice de Vulnerabilidad Territorial de barrios y distritos de Madrid y Ranking de Vulnerabilidad (2018)” [29]. 1—Centro; 2—Arganzuela; 3—Retiro; 4—Salamanca; 5—Chamartín; 6—Tetuán; 7—Chamberí; 8- Fuencarral El Pardo; 9—Moncloa-Aravaca; 10—Latina; 11—Carabanchel; 12—Usera; 13—Puente de Vallecas; 14 Moratalaz; 15—Ciudad Lineal; 16—Hortaleza; 17—Villaverde; 18—Villa de Vallecas; 19—Vicálvaro; 20—San Blas-Canillejas; 21—Barajas.

These distinct geographical and social differences prevail today. However, they are now analyzed according to the broader and more precise concept of “vulnerability” (Figure 1). Therefore, it was decided to conduct a space-time analysis by district. It should be noted that in this type of analysis, focused on smaller areas, such as districts, neighborhoods, or intersections, it is difficult to conduct certain estimation variables and methods [30,31,32]. However, these small-scale analyses can reveal other effects which would be more difficult to assess in wider areas [33].

### 2.1. Dependent Variables

The dependent variable in this analysis is the annual number of victims (any person killed or injured (minor or serious) as a result of a traffic accident) between 2013 and 2019 (T = 7 years), in the 21 districts of Madrid (N × T = 147). The accident rate data were downloaded from the open data portal of the City Council of Madrid [34]. The downloaded files register the accidents with injuries or damage to municipal assets, classified by district.

The accidents are categorized in the data according to the severity of the injuries: “Serious” (any person injured in a traffic accident and has to be admitted to hospital for more than 24 h), “Minor” (any person injured in a traffic accident to whom the definition of seriously injured does not apply), and “Death” (any person who, as a result of a traffic accident, dies at the scene or in the following thirty days) [35].

### 2.2. Independent Variables

The principal independent variable used in this study is a fictitious variable that varies over time, and which indicates the availability of the services of Uber and Cabify in Madrid. This binary variable takes the values of 1 and 0 to indicate the presence and absence of these services, respectively. In order to determine the year when the Uber and Cabify services began to operate in Madrid, in addition to consulting the information drawn from the general media, other variables were considered, such as: the revenue of Uber System Spain S.L and Maxi Mobility Spain S.L (Cabify, Madrid, Spain), and the number of CDV licenses in the Region of Madrid, which indicate the real deployment of these platforms in the Spanish capital [36].

**Figure 2:**
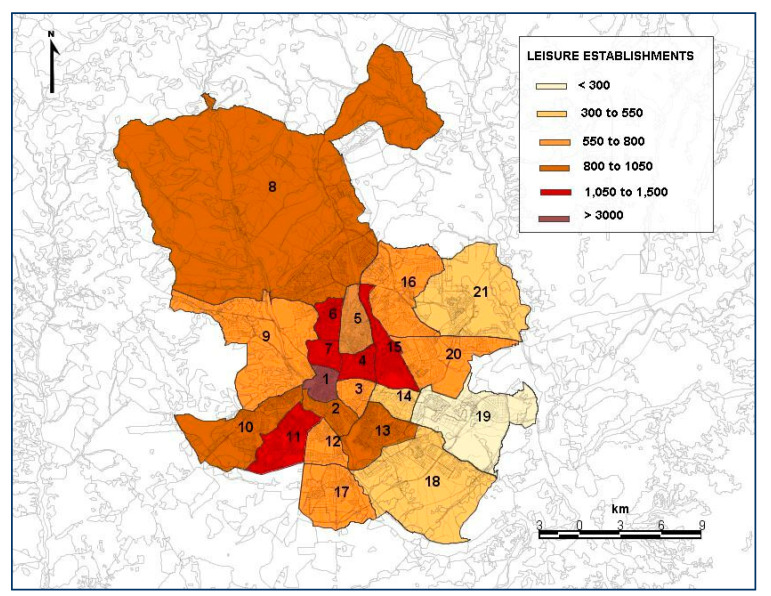
Average number of leisure establishments in the districts of Madrid analyzed in the period 2013–2019. Source: Own elaboration, based on the data drawn from the statistical information department of the City Council of Madrid [38]. 1—Centro; 2—Arganzuela; 3—Retiro; 4—Salamanca; 5—Chamartín; 6—Tetuán; 7—Chamberí; 8- Fuencarral El Pardo; 9—Moncloa-Aravaca; 10—Latina; 11—Carabanchel; 12—Usera; 13—Puente de Vallecas; 14 Moratalaz; 15—Ciudad Lineal; 16—Hortaleza; 17—Villaverde; 18—Villa de Vallecas; 19—Vicálvaro; 20—San Blas-Canillejas; 21—Barajas.

Dumbaugh and Rae [37] analyzed how the urban nature (specifically, the use of the land and configuration of the street network) could influence the incidence of injury and death due to traffic-related collisions. They also found that certain types of commercial uses were related to a greater risk of accident. For this reason, the number of leisure establishments available in each district analyzed has been considered (Figure 2).

**Figure 3:**
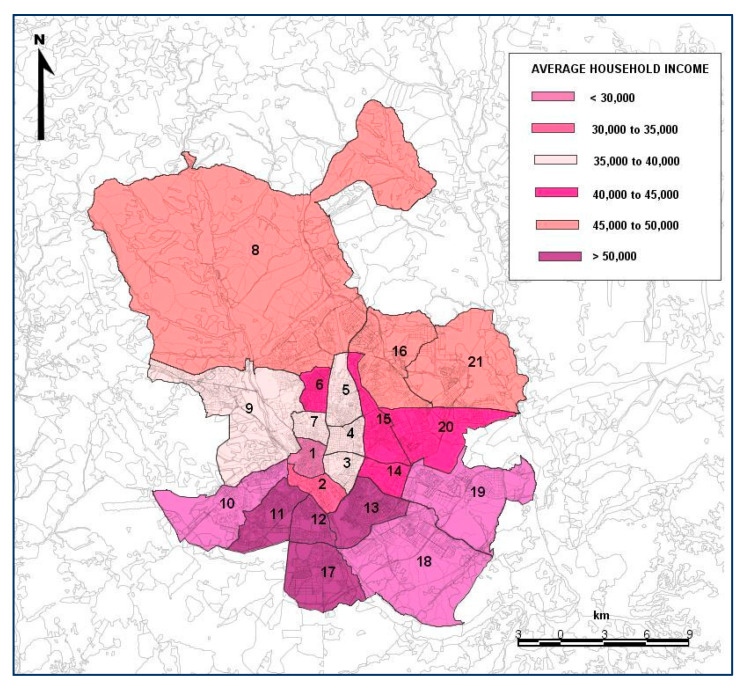
Average household income in the districts of Madrid analyzed in the period 2013–2019. Source: Own elaboration, based on the data drawn from the statistical information department of the City Council of Madrid [40,41]. 1—Centro; 2—Arganzuela; 3—Retiro; 4—Salamanca; 5—Chamartín; 6—Tetuán; 7—Chamberí; 8—Fuencarral El Pardo; 9—Moncloa-Aravaca; 10—Latina; 11—Carabanchel; 12—Usera; 13—Puente de Vallecas; 14—Moratalaz; 15—Ciudad Lineal; 16—Hortaleza; 17—Villaverde; 18—Villa de Vallecas; 19—Vicálvaro; 20—San Blas-Canillejas; 21—Barajas.

Socioeconomic factors play a very important role in road safety. Many of the studies published have analyzed the influence of economic conditions on traffic accidents with variables such as the level of unemployment. The results obtained show a direct relationship between accidents and the economic level of a country, with less accidents occurring during times of recession and increasing during periods of economic growth [39]. Therefore, this study also takes into consideration the socioeconomic status of each district of Madrid (Figure 3). This variable is an indicator calculated based on the average income per household and district, following the methodology used in the report conducted by the “Metodología para la elaboración del Índice de Vulnerabilidad Territorial de barrios y distritos de Madrid y Ranking de Vulnerabilidad (2018)” [29,36].

**Figure 4:**
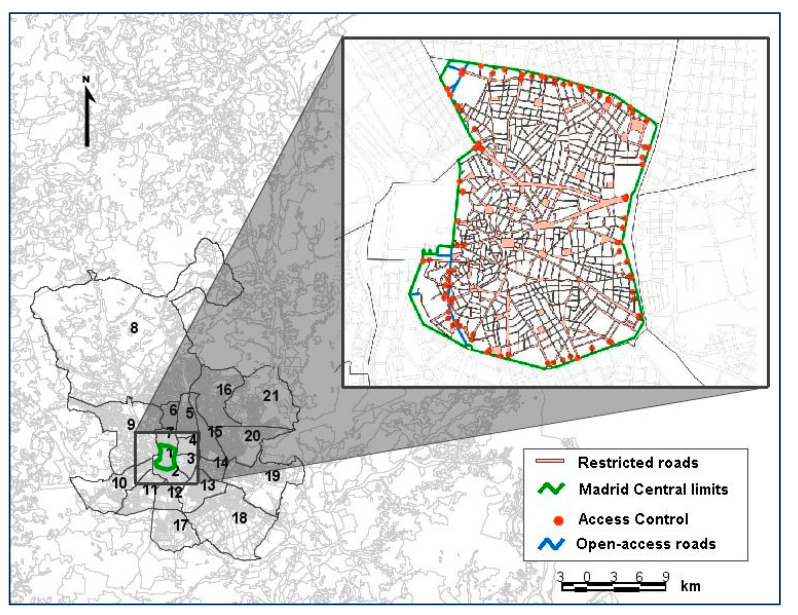
Residential priority area (RPA) or low emission zone (LEZ) in the downtown district. Source: Own elaboration, based on the data drawn from the Geoportal of the city council of madrid [43]. 1—Centro; 2—Arganzuela; 3—Retiro; 4—Salamanca; 5—Chamartín; 6—Tetuán; 7—Chamberí; 8—Fuencarral El Pardo; 9—Moncloa-Aravaca; 10—Latina; 11—Carabanchel; 12—Usera; 13—Puente de Vallecas; 14—Moratalaz; 15—Ciudad Lineal; 16—Hortaleza; 17—Villaverde; 18—Villa de Vallecas; 19—Vicálvaro; 20—San Blas-Canillejas; 21—Barajas.

On 30 November 2018, the residential priority area (RPA) or low emission zone (ZBE) was inaugurated in Madrid’s Central District, which restricts access to the most polluting vehicles (Figure 4) with the objectives of: improving road safety; the necessary, orderly, and respectful coexistence between the different modes of transportation; the protection of human health through substantial improvement of air quality; promoting public transport and collective public intermodal transport, pedestrian, and cycling mobility; the development of electric and cleaner mobility and car sharing; to organize the different uses of urban roads and public spaces [42]. Therefore, a binary indicator was included to know if traffic restrictions were present in the downtown district in a given year (0, no traffic restrictions in the downtown district; 1, with traffic restrictions in the downtown district).

**Figure 5:**
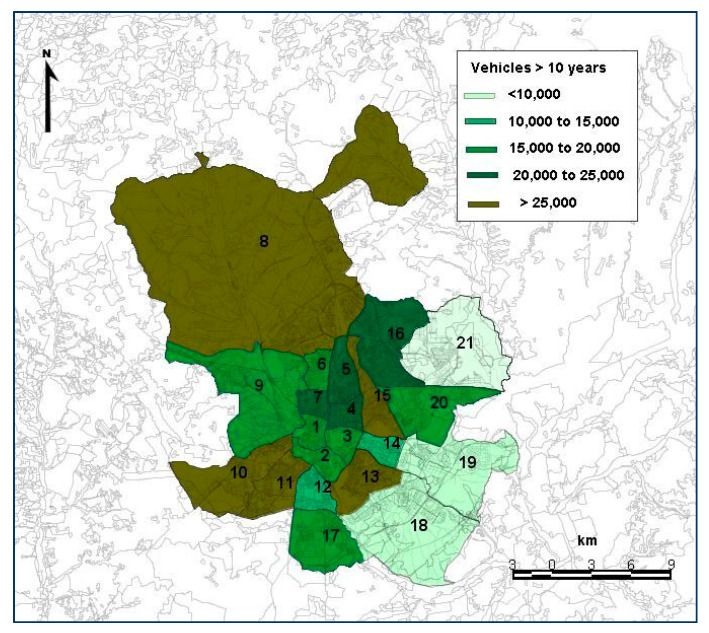
Average number of vehicles aged 10 years or more in the period 2013–2019 in each district of Madrid. Source: Own elaboration, based on the data drawn from the statistical information department of the City Council of Madrid [45]. 1—Centro; 2—Arganzuela; 3—Retiro; 4—Salamanca; 5—Chamartín; 6—Tetuán; 7—Chamberí; 8—Fuencarral El Pardo; 9—Moncloa-Aravaca; 10—Latina; 11—Carabanchel; 12—Usera; 13—Puente de Vallecas; 14—Moratalaz; 15—Ciudad Lineal; 16—Hortaleza; 17—Villaverde; 18—Villa de Vallecas; 19—Vicálvaro; 20—San Blas-Canillejas; 21—Barajas.

In aaddition, a variable related to the age of the vehicle fleet (the number of vehicles aged 10 years or more) has also been included for each district analyzed (Figure 5). Few models analyze the influence of the age of the vehicles on accidents. Specifically, Fontaine [44], using French data, revealed that the risk of accident of passenger cars aged ten years or more was 60% higher than that of those aged three years or less.

In Table 1, a descriptions of the variables considered in this analysis has been provided.

In this study, a logarithmic transformation was applied “log(X)” to optimize vehicles ≥10 years per area variable in order to eliminate problems such as heteroscedasticity and to obtain variance-stabilizing transformations of variables in the statistical model [33].

There are additional factors, such as driver psychophysical conditions which can be considered in modeling injury severity. At-risk drivers (including drink drivers) are at greater risk of suffering a very serious accident, with public holidays and weekends being the days of the year with the highest probability of suffering an accident. However, the presence of alcohol or drugs was not considered in this study because the authors of this paper have already analyzed, in previous research, the influence of Uber and Cabify in fatal and serious accidents with the presence of alcohol or drugs and those occurring on weekends or holidays [36,46]. Other factors such as driving fatigue, sleep, continuous hours of driving could not be considered in this study because the data relevant to such factors were not available in the data set consulting.

In the case of weather conditions, they have not been considered because we are analyzing the number of injuries and deaths in urban areas and, in this case, it is easier to foresee a dangerous situation due to bad weather, unlike on the road, where there is a greater probability of suffering a traffic accident due to rain or fog.

Therefore, the scope of this study was limited to investigating the effects of socioeconomic characteristics and vehicle attributes on crash severity. Nevertheless, due to such omitted variables, crashes within districts are likely to be correlated and similar in terms of unobservable characteristics. This could be a potential source of unobserved district-specific heterogeneity. Neglecting to consider such an important issue may cause biases in parameter estimates and model results.

### 2.3. Statistical Methods

In order to analyze the implications of the ride-hailing services on the number of victims caused by traffic accidents, each of the districts of Madrid were observed in several periods of time (from 2013 to 2019). To do this, the information is presented as panel data. An advantage of using panel data in econometric estimations is that greater precision in the estimated parameters can be obtained, since there is a higher number of observations because the cross-sectional data are combined with the time series data. However, this benefit is only obtained by correcting for any type of serial correlation in the observations related to any individual [47].

Furthermore, in order to explain the behavior and evolution of the frequency of traffic accidents, the statistical models most used are the Poisson and negative binomial (NB) models, as accidents are discreet, non-negative, and often infrequent and random [48]. However, there are situations in which the observed count data does not fulfil all of the assumptions of the Poisson regression model. One of the most common situations found in practice is that the individual counts can exhibit more variability than expected of the Poisson model, which assumes that the mean and conditional variance are equal (it should be remembered that the Poisson distribution has a λ_i_, parameter which characterizes the mean and variance of the distribution), a condition known as equidispersion. If the variance is larger than the mean, there is overdispersion and the model would not be appropriate [49].

Several previous studies have indicated that traffic accident data were significantly overdispersed [50,51]. This occurs for two primary reasons in cross-sectional data. First, there may be individual differences in responses that are not accounted for by the regression model. This problem commonly occurs if an important predictor is omitted from the model. Second, each count that occurs for an individual may not be an independent event as is assumed by the Poisson distribution. This situation is known as *contagion* or *state dependence*. In addition, one shortcoming of the Poisson regression model is that it does not contain an error (disturbance) term that fully parallels the error term found in an ordinary least squares (OLS) regression equation. Often there is additional heterogeneity among individuals that is not accounted for by the predictors in the model and the Poisson error function alone, which results in over dispersion. This can be overcome by introducing the negative binomial model which accounts for over dispersion by assuming that there are unexplained variability among individuals who have the same predicted value. This additional unexplained variability between individuals leads to larger variance (than expected by the Poisson distribution) in the overall outcome distribution but has no effect on the mean. This additional variability is conceptually similar to the inclusion of an error term in normal linear regression. The Poisson model assumes that the outcomes for all individuals with the same values on the predictors are samples from a single Poisson distribution with a given mean. However, the negative binomial model allows the observations of individuals with the same values on the predictors to be modeled by Poisson distributions with different mean parameters [49].

To this end, the negative binomial model uses another standard (although less familiar) probability distribution, the gamma distribution, to represent the distribution of means [52]. In this model, the error function is a mixture of two different probability distributions, Poisson and gamma distributions [49].

A useful way to motivate the model is through the introduction of latent heterogeneity in the conditional mean of the Poisson model [53]:

*E*[*y_i_*|*x_i_*, *ε_i_*] = exp (*α* + *x_i_*′*β* + *ε_i_*) = *h_i_ λ_i_* (1)

where *λ_i_ =* exp (*α + x_i_*′*β*), *x_i_*′ is a vector of covariates for segment (district) *i,; β_i_* is an estimable regression coefficients; *y_i_* and *E*[*y_i_*|*x_i_*, *ε_i_*] are the observed and predicted number of head-on crashes on road segment (district) *i*, respectively; and *h_i_ =* exp *(ε_i_)* is assumed to have a one parameter gamma distribution, G*(θ,θ),* with mean 1 and variance *1/θ = k*; therefore:

(2) $$f\left(h_{i}\right)=\frac{\theta^{\theta }\exp\left(-\theta h_{i}\right)h_{i}^{0-1}}{\Gamma \left(\theta \right)}h_{i}\geq 0,\theta >0_{\;}$$

After integrating *h_i_* out of the join distribution, we obtain the marginal negative binomial distribution:

(3) $$Prob\;\left[Y=y_{i}|x_{i}\right]=\frac{\Gamma \left(\theta \;+y_{i}\right)\cdot r_{i}^{\theta }{\left(1-r_{i}\right)}^{y_{i}}}{\Gamma \left(1+y_{i}\right)\Gamma \left(\theta \right)}y_{i}=0,1,\ldots ,\theta >0,\;r_{i}=\theta /{\left(\theta +\lambda_{i}\right)}_{\;}$$

The conditional mean of the outcome, given the values of the predictors, is identical for the Poisson model and the negative binomial model:

(4) $$E\;\left[y_{i}|x_{i}\right]=\lambda_{i}$$

In contrast, the conditional variance of the outcome will be larger in the negative binomial model than in the Poisson model. The variance for the negative binomial model is given by:

(5) $$\mathrm{Var}\;\left[y_{i}|x_{i}\right]=\lambda_{i}\left[1+\left(1/\theta \right)\lambda_{i}\right]=\lambda_{i}\left[1+k\lambda_{i}\right]$$

where *k =* Var[*h_i_*].

The parameters of the negative binomial model are estimated by standard maximum likelihood methods through maximizing the logarithm of likelihood function (Equation (3)).

The superiority of the negative binomial model over the Poisson regression model depends on the value of dispersion parameter *k*. If *k* is not statistically different from zero, the negative binomial model reduces to the Poisson model [54].

#### Heterogeneity in Panel Data

A basic assumption of standard Poisson and NB models, such as likelihood-based models, is that observations are independent. However, this assumption is often violated for road crash data in which observations coming from a same group are correlated spatially or/and temporally. Such correlations arise from unobserved factors which may exist in same groups. For example, crash counts may be independent across road segments, but those occurring on a specific road segment over successive time periods are likely to be serially correlated. Ignoring such possible correlations violates fundamental assumption of “independence of errors” in standard count models, and also underestimates standard errors of model parameters.

Some recent studies have attempted to account for unobserved heterogeneity using random effect (RE) terms representing between-site variances, which are consistent for extremes in the same site but vary between different sites [55,56,57,58,59,60]. This approach, while representing an improvement over models that do not account for unobserved heterogeneity, still has limitations. Some of these limitations have been addressed by introducing random parameters to a Bayesian hierarchical univariate extreme value approach for traffic conflict-based crash estimation [61,62].

Another way to account for within-site correlation (or heterogeneity) as well as between-site unobserved heterogeneity is to treat road crashes as panel data and apply a random-effect NB model.

The random-effects negative binomial (RENB) model assumes that the within-segment heterogeneity is uncorrelated with the explanatory variables. If this assumption does not hold, a fixed-effect (FE) specification should be used. The selection between FE and RE models is based on the Hausman test, which determines which model is more appropriate than the other [63]. This test is implemented through the statistics software package STATA version 16, which was used to develop and select the appropriate statistical model for this analysis. If the test is insignificant, the RE model is preferred over the FE model, implying that there is no correlation between independent variables and random effects [64]. In the context of road safety, there are some studies which have applied random-effect models [65,66,67]. For example, Chin and Quddus [68] used a RENB model to investigate variables contributing to traffic accidents at signalized intersections in Singapore. Kweon and Kockelman [64] applied fixed- and random-effect models along with several other count models to examine safety effects of speed limit changes on high-speed highways. Shankar et al. [69] identified the random effects negative binomial (RENB) model as being more appropriate for modeling median crossover crash frequencies in relation to geometric and traffic variables in Washington State. All these studies have found the RENB model suitable for the variables (i.e., geometric and traffic) which are likely to have location specific effects. Moreover, from an analytical viewpoint, RENB models offer advantages in terms of model transferability and updating [48,63,70].

Let *Yit* be the number of head-on crashes on district *i* in year *t*, the framework for random-effects negative binomial (RENB) model is given as:

(6) $$E\left(Y_{it}\right)=\exp{\left(\beta X_{it}+u_{i}+\varepsilon_{it}\right)}_{\;}$$

where *E*(*Y_it_*) represents the expected number of accidents in the *i*^th^ district in year *t*; *X_it_* is the vector of explanatory variables; *β* is the vector of regression parameters; *ε_it_* is the vector of residual errors; *u_i_* represents the random effect for the *i*^th^ location; and exp(*u_i_*) is gamma distributed with mean 1 and variance α_i_, where α_i_ is the parameter of overdispersion in the negative binominal model.

The RENB model allows the overdispersion parameter to vary randomly from district to district, such that *1/(1 + α_i_)* follows a Beta (r, s) distribution [69,71].

In light of the above, the model that is the most appropriate, and therefore, selected for this study is the random effects negative binomial (RENB), as available crash data have a panel structure, and the variables are likely to have location specific effects.

### 2.4. Goodness-of-Fit of the Model

The goodness-of-fit of the model was tested and compared using the McFadden pseudo-R-squared value [72], estimated as follows:

(7) $$R^{2}=1-\frac{LL\left(\beta \right)}{LL\left(C\right)}$$

where *LL(β*) is the log-likelihood value of the full model, and *LL(C)* is log-likelihood value of the constant only model.

Another measure, $R_{\alpha }^{2}$, proposed by Miaou et al. [73] was deployed in this study to determinate how well the variance of data is captured by the model relative to a fundamental model with no variables [74]. This measure takes the NB dispersion parameter and is estimates as follows:

(8) $$R_{\alpha }^{2}=1-\frac{\alpha }{1+\alpha_{\max}}$$

where α is the estimated over dispersion parameter for the selected model and α_max_ is the estimated overdispersion parameter for the fundamental model containing only constant term [75].

To compare the models, two information criteria were used: the Akaike information criterion (AIC) and the Bayesian information criterion (BIC). The AIC and BIC are defined as follows:

(9) AIC=−2LL+2P

(10) BIC=−2LL+P(ln(n))

where *LL* is the logarithm of the maximum likelihood estimation for each model, *P* is the number of model parameters, and *n* is the number of observations (*n* = 147).

A model with the lowest AIC and BIC values is preferred. To decide whether there is a statistically significant difference between two models, Hilbe’s AIC and Raftery’s BIC rule-of-thumb criteria were adopted in this study [76,77], as shown in the table below (Table 2):

In this case study (*n* = 147), if the difference in the AIC value is greater than 10, then the model with lower AIC is favored over another.

## 3. Results and Discussion

### 3.1. Goodness-of-Fit Model and Selection

Table 3 provides a brief description and statistical summary of the variables used in the analysis of the traffic accident victims. One of the principal input characteristics is that the mean is higher than the standard deviation in each of the dependent variables analyzed. These figures imply that the negative binomial regression model is more appropriate than the Poisson regression model.

Table 4 represents the Pearson correlation coefficient matrix between all of the variables considered for the model. The results show that none of the variables are correlated with others, as the correlation coefficients are lower than ±0.7.

Table 5 shows the results of the comparison of the random effects negative binomial (RENB) and negative binomial (NB) models. The results show that the RENB model gives rise to a significantly better log-likelihood in the convergence than the NB model.

Furthermore, the RENB model improves the overall fit (R^2^ = 0.227 total victims, R^2^ = 0.217 minor injuries, R^2^ = 0.118 serious injuries and deaths) as compared with the NB model (R^2^ = 0.038 total victims, R^2^ = 0.039 minor injuries, R^2^ = 0.043 serious injuries and deaths). The results of the likelihood ratio test vs. pooled test indicate that the panel estimation is significant as compared with the pooled estimation.

The results also show that both the AIC and the BIC give the RENB an advantage over the NB model. In terms of the AIC, the RENB model has lower values. The superiority of the RENB model is also supported by the BIC.

Furthermore, the Hausman specification test is not significant; therefore, we fail to reject the null hypothesis of equal estimates, and therefore the most efficient estimator is that of random effects.

### 3.2. Main Analysis

Table 6 presents the results of the three dependent variables studied. The “ride-hailing services” variable is significant in the three cases with a negative coefficient in Model 3, which indicates that the introduction of these services is associated with a reduction of around 25% of the number of seriously injured and deaths. However, the number of total victims increased by around 3% and the number of minor injuries increased by around 5%.

These results are compatible with those obtained by Kirk et al. [18], who analyzed the involvement of these services in the deaths and injuries caused by traffic accidents in Great Britain and observed a reduction of 9% in serious injuries after the arrival of Uber.

There are several explanations for why the availability of Uber and Cabify appears to be associated with a significant reduction in serious injuries and death. For instance, Uber and Cabify may be an alternative form of transportation used by the types of risky drivers, including drink drivers, which are at greater risk of getting in very serious accidents. According to a report issued by Uber and Mothers against Drunk Driving (MADD, Irving, TX, USA), young people prefer to use this service rather than driving their own vehicles if they plan to consume alcohol, because “when people have more options, they make better and safer choices”. The results of the study are compatible with other data, for example, after the UberX service began operating in cities in California, the monthly accidents related to alcohol dropped by 6.5% among drivers under the age of 30 [19]. A study on California found that the advent of UberX resulted in a 3.6–5.6% decrease in the rate of motor vehicle homicides per quarter [21]. Morrison et al. [25] found a relationship between the 62% reduction in the accident rate related to alcohol and the implementation of ride-hailing platforms in the city of Portland (Oregon). In the case of New York, a reduction of 25–35% (around 40 collisions per month) was observed in the collision rate related to alcohol in the districts of Manhattan, Bronx, Brooklyn and Queens [23]. In Madrid, the implementation of Uber and Cabify services was associated with a reduction in accidents with serious injuries or fatalities and involving alcohol and drugs [36,46].

However, Uber and Cabify may be a substitute for taxis and other forms of publics transportation but not a substitute for moderately risky driving (i.e., the type of drivers who get into accidents involving “slight” injuries). Indeed, research reveals that the advent of Uber in the United States has been associated with a decline in the use of bus [78,79] and light rail, and may serve as a complementary mode for commuter rail (3% net increase in use). In addition, ride-hailing users were asked which transportation alternatives they would have used for the trips that they currently make using Uber and Lyft. Based on the frequency of ride-hailing use weighted data, a majority (61%) of trips would have not been made at all, or by walking, biking, or transit; 39% of trips would have been made by car (drive alone, carpool, or taxi). Using data unweighted by frequency of ride-hailing use, 49% of ride-hailing trips were likely to have not been made at all, or by walking, biking, or transit [80].

Therefore, if former public transit users are the predominant Uber customer rather than moderately risky drivers, the number of moderately risky drivers on the road would not decline and, consequently, the number of cars and even the number of risky drivers (e.g., such as those who are sleep deprived) on the road may even increase, which may, then, lead to increases in slight injuries [18], especially in a dense city like Madrid.

However, these findings are based on results obtained in U.S. cities, and may not be reflected in the case of Madrid. Therefore, we recommend for future research an in-depth analysis of the use of these services and their influence on travel behavior in the municipality of Madrid.

With respect to the socioeconomic status variable, this variable indicates that fatalities and serious injuries have been reduced in the most vulnerable districts of Madrid. This coincides with the results of Flor et al. [36] who indicated that, since Uber and Cabify started operating, accidents occurring on weekends and public holidays with serious injuries or fatalities also decreased in the most vulnerable districts.

With regard to the leisure establishments, this variable is statistically significant with a positive coefficient in Models 2 and 3, indicating that there is a close relationship between the agglomerations of leisure establishments (restaurants, pubs, theatres, cinemas, etc.) and the number of total victims and minor injuries.

The same is the case with the variable “vehicles > 10 years”, which is significant with a positive coefficient in the three models, indicating that the age of the vehicle affects the number of victims per accident. This result coincides with that obtained by Anderson and Searson [60] who observed that the risk of accident seemed to be the lowest when vehicles were two years old, and increased as they aged. In an analysis carried out in Australia and New Zealand, Smith [81] observed that the oldest vehicles were over-represented in the deaths of their occupants during the period 2012–2016. The result of the study conducted by Török [82] also showed that the age of the vehicle was strongly related to the occurrence of accidents. This is due, mainly, to the fact that more modern vehicles incorporate elements that influence the driver before a situation of risk arises, such as electronic stability control (ESP) and traction control system (TCS), anti-lock braking system (ABS), etc. Furthermore, modern passenger cars are able to better protect their occupants as shown in the different assessment tests, such as EuroNCAP, which have shown that a modern vehicle was much safer than one that is ten years old [83].

The results obtained with respect to “Madrid Central” imply that the entry into force of Sustainable Mobility Ordinance, 5 October 2018 [42] has contributed to a reduction in serious injuries and fatalities in District 1.

## 4. Conclusions

The results obtained in this study suggest that the arrival of Uber and Cabify to the municipality of Madrid could be associated with a reduction in the number of serious injuries and deaths in traffic accidents. However, it is important to note that this study has a limitation, which is common to all academic studies analyzing the association of the introduction of these services with injury and death due to traffic accidents. This limitation is related to the lack of information on an individual level, with respect to the trips, the drivers, and the users of these services. Therefore, it is not possible to examine on an individual level, whether drivers who have consumed alcohol or other high-risk drivers make use of these services. Similarly, we do not have information about the specific risk profile of Uber drivers (tiredness, hours of driving, etc.).

In addition, there are other limitations in this study merit further study. Firstly, the driver psychophysical characteristics that affect driving violations should be further explored to refine the influencing factors; secondly, the research results can be extended to improve road safety analysis [61,62].

Road safety is one of the principal problems of today’s society. So much so, that the 2030 Agenda for Sustainable Development establishes two objectives: Target 3.6 pledges to halve by 2020 “the number of global deaths and injuries from road traffic accidents” and Target 11.2 calls on providing, by 2030, “access to safe, affordable, accessible and sustainable transport systems for all, improving road safety”. To do this, as previously mentioned at the beginning of this study, as well as road safety, the influence of these services on traffic congestion and CO_2_ emissions should also be studied in those cities where they operate. In this way, depending on the results obtained, the cities can adopt appropriate measures with respect to the regulation of these services.

## Figures and Tables

**Table 1:** Variables. Source: own research.

Variables	Definition
Total victims	Number of people who died or sustained injuries (minor or serious) as a result of a traffic accident.
Serious injuries and deaths	Number of people injured in a traffic accident admitted to hospital for more than 24 h and the number of people who, due to traffic accidents, died at the scene or in the following thirty days.
Minor injuries	Number of people injured in a traffic accident to whom the definition of serious injury does not apply.
Ride-hailing services	Year dummy variables: 1, Uber or Cabify presence 0, neither Uber nor Cabify
Leisure establishments	Variable that measures the number of establishments dedicated to the catering, leisure, and entertainment activities per hectare
Socioeconomic status	Indicator calculated based on the average income per household and district
Madrid Central	Year and district dummy variables: 1, traffic restrictions in the Downtown District 0, no traffic restrictions in the Downtown District
Vehicles ≥ 10 years	Variable measuring the number of vehicles 10 years old or older per hectare

**Table 2:** Significance levels for AIC and BIC. Source: Raftery, 1995 and Hilbe, 2011.

∆AIC for Models A and B	Result if A < B	∆BIC for Models A and B	Result if A < B
<0.0 and ≤2.5	No difference	<0.0 and ≤2.0	Weak difference
<2.5 and ≤6.0	Prefer A if *n* > 256	<2.0 and ≤6.0	Positive difference
<6.0 and ≤9.0	Prefer A if *n* > 64	<6.0 and ≤10.0	Strong difference
10+	Prefer A	10+	Very strong difference

**Table 3:** Descriptive statistics. Source: own research.

	Obs.	Mean	Standard Deviation	Minimun	Maximun
**Dependent variables**					
Total Victims	147	609.3401	230.4578	146	1127
Minor injuries	147	564.0136	215.4313	130	1065
Serious injuries and deaths	147	45.32653	19.64206	2	80
**Independent variables**					
Ride-hailing Services	147	0.5714	0.4966	0	1
Socio-economic Status (%)	147	0.7326	0.0105	0.7090	0.7465
Leisure Establishments/area	147	1.0480	1.3243	0.0325	6.2878
Madrid Central	147	0.0068	0.0824	0	1
Log Veh ≥ 10 years/area	147	1.0825	0.4966	0.0324	1.6215

**Table 4:** Pearson’s correlation coefficients between variables. Source: own research.

	Ride-Hailing Services	Socio-Economic Status (%)	Leisure Establishments/Area	Madrid Central	Log Veh > 10 Years/Area
Ride-hailing Services	1.0	0	0.0746	0.0717	0.0779
Socio-economic Status (%)	0	1.0	−0.0511	0.0399	−0.0228
Leisure Establishments/area	0.0746	−0.0511	1.0	0.3286 ***	0.6007 ***
Madrid Central	0.0717	0.0399	0.3286 ***	1.0	0.0719
Log Veh > 10 years/area	0.0779	−0.0228	0.6007 ***	0.0719	1.0

*** Significant at 0.001 level.

**Table 5:** Results of the fitted models. Source: own research.

	Model 1 Total Victims		Model 2 Minor Injuries		Model 3 Serious Injuries and Deaths	
Models	NB	RENB	NB	RENB	NB	RENB
Log-likelihood at converge	−1019.301	−1019.301	−1008.903	−1008.903	−653.5397	−653.5397
Log-likelihood with constant only	−980.3115	−787.8321	−969.2651	−789.5833	−625.2342	−576.6345
McFadden pseudo R^2^	0.038	0.227	0.039	0.217	0.043	0.118
AIC	1974.623	1591.664	1952.53	1595.167	1264.468	1169.269
BIC	1995.556	1615.668	1973.463	1619.09	1285.401	1193.193
Dispersión parameter, α (95% CI)	0.112 (0.089–0.141)		0.113 (0.09–0.142)		0.138 (0.104–0.182)	
$R_{\alpha }^{2}$	0.9056		0.91		0.89	
/ln_r		2.584 (7.28)		2.538 (7.41)		2.807 (8.06)
/ln_s		2.689 (7.16)		2.890 (7.82)		3.130 (8.40)
LR test vs. pooled: chibar2(01)		356.08		330.52		78.25
Prob≥chibar2		0.000		0.000		0.000
Hausman Test: chi2(8) Prob > chi2		10.68 0.0580		8.42 0.1343		1.36 0.9285

**Table 6:** Results of the estimated model (z-statistics in parentheses). Source, own research.

	Dependent Variables		
Independent Variables	Model 1 Total Victims	Model 2 Minor Injuries	Model 3 Serious Injuries and Deaths
Ride-hailing services	0.0319 * (2.23)	0.0517 *** (3.35)	−0.253 *** (−6.17)
Socioeconomic status (%)	4.477 (0.67)	1.703 (0.26)	−13.27 * (−2.00)
Leisure Establishments/area	0.0741 * (2.10)	0.0773 * (2.11)	0.0679 (1.09)
Log Veh > 10 years/area	0.385 *** (3.98)	0.409 *** (4.04)	0.445 ** (2.69)
Madrid Central	−0.0681 (−1.21)	−0.0239 (−0.39)	−1.177 *** (−3.30)
Log-likelihood	−787.8321	−789.5833	−576.6345
Wald chi2	80.67	102.35	58.97
Prob > chibar	0.000	0.000	0.000
/ln_r	2.584 *** (7.28)	2.538 *** (7.41)	2.807 *** (8.06)
/ln_s	2.689 *** (7.16)	2.890 *** (7.82)	3.130 *** (8.40)
LR test vs. pooled: chibar2(01)	148.74	330.52	78.25
Prob ≥ chibar2	0.000	0.000	0.000

*** Significant at 0.001 level, ** Significant at 0.01 level, * Significant at 0.05 level.

## Data Availability

Publicly available datasets were analyzed in this study. This data can be found here: Traffic accidents in the City of Madrid: https://datos.madrid.es/portal/site/egob/menuitem.c05c1f754a33a9fbe4b2e4b284f1a5a0/?vgnextoid=7c2843010d9c3610VgnVCM2000001f4a900aRCRD&vgnextchannel=374512b9ace9f310VgnVCM100000171f5a0aRCRD&vgnextfmt=default (accessed on 18 August 2020); Leisure Establishments: http://www-2.munimadrid.es/CSE6/control/seleccionDatos?numSerie=4020400031 (accessed on 18 December 2020); Vehicles ≥ 10 years: https://www.madrid.es/portales/munimadrid/es/Inicio/El-Ayuntamiento/Estadistica/Anuario-Estadistico-Municipal-desde-2004-/?vgnextfmt=default&vgnextoid=7e1e63af4fe46310VgnVCM2000000c205a0aRCRD&vgnextchannel=8156e39873674210VgnVCM1000000b205a0aRCRD (accessed on 15 August 2021); Household income: https://www.madrid.es/portales/munimadrid/es/Inicio/El-Ayuntamiento/Estadistica/Areas-de-informacion-estadistica/Economia/Renta/Urban-Audit/?vgnextfmt=default&vgnextoid=6d40393c7ee41710VgnVCM2000001f4a900aRCRD&vgnextchannel=ef863636b44b4210VgnVCM2000000c205a0aRCRD (accessed on 1 April 2020); https://www.ine.es/jaxiT3/Datos.htm?t=31097 (accessed on 1 April 2020).
